# Cell-selective knockout and 3D confocal image analysis reveals separate roles for astrocyte-and endothelial-derived CCL2 in neuroinflammation

**DOI:** 10.1186/1742-2094-11-10

**Published:** 2014-01-21

**Authors:** Debayon Paul, Shujun Ge, Yen Lemire, Evan R Jellison, David R Serwanski, Nancy H Ruddle, Joel S Pachter

**Affiliations:** 1 Department of Cell Biology, Blood–brain Barrier Laboratory, 263 Farmington Ave., Farmington CT 06030, USA; 2 Department of Immunology, University of Connecticut Health Center, Farmington CT 06030, USA; 3 Department of Physiology and Neurobiology, University of Connecticut, Storrs CT 06269, USA; 4 School of Public Health, Yale University, New Haven CT 06520, USA

**Keywords:** Astrocytes, Blood–brain barrier, Brain microvascular endothelial cells, CCL2, Claudin-5, Central nervous system, Experimental autoimmune encephalomyelitis, Neuroinflammation, 3D confocal imaging

## Abstract

**Background:**

Expression of chemokine CCL2 in the normal central nervous system (CNS) is nearly undetectable, but is significantly upregulated and drives neuroinflammation during experimental autoimmune encephalomyelitis (EAE), an animal model of multiple sclerosis which is considered a contributing factor in the human disease. As astrocytes and brain microvascular endothelial cells (BMEC) forming the blood–brain barrier (BBB) are sources of CCL2 in EAE and other neuroinflammatory conditions, it is unclear if one or both CCL2 pools are critical to disease and by what mechanism(s).

**Methods:**

Mice with selective CCL2 gene knockout (*KO*) in astrocytes (*Astro KO*) or endothelial cells (*Endo KO*) were used to evaluate the respective contributions of these sources to neuroinflammation, i.e., clinical disease progression, BBB damage, and parenchymal leukocyte invasion in a myelin oligodendrocyte glycoprotein peptide (MOG_35-55_)-induced EAE model. High-resolution 3-dimensional (3D) immunofluorescence confocal microscopy and colloidal gold immuno-electron microscopy were employed to confirm sites of CCL2 expression, and 3D immunofluorescence confocal microscopy utilized to assess inflammatory responses along the CNS microvasculature.

**Results:**

Cell-selective loss of CCL2 immunoreactivity was demonstrated in the respective *KO* mice. Compared to wild-type (*WT*) mice, *Astro KO* mice showed reduced EAE severity but similar onset, while *Endo KO* mice displayed near normal severity but significantly delayed onset. Neither of the *KO* mice showed deficits in T cell proliferation, or IL-17 and IFN-γ production, following MOG_35-55_ exposure *in vitro*, or altered MOG-major histocompatibility complex class II tetramer binding. 3D confocal imaging further revealed distinct actions of the two CCL2 pools in the CNS. *Astro KO*s lacked the CNS leukocyte penetration and disrupted immunostaining of CLN-5 at the BBB seen during early EAE in *WT* mice, while *Endo KO*s uniquely displayed leukocytes stalled in the microvascular lumen.

**Conclusions:**

These results point to astrocyte and endothelial pools of CCL2 each regulating different stages of neuroinflammation in EAE, and carry implications for drug delivery in neuroinflammatory disease.

## Background

The chemokine CCL2 (formerly called Monocyte Chemoattractant Protein-1, *MCP-1*) has long been established as a critical mediator of inflammation within and outside the central nervous system (CNS), and stimulates extravasation of mononuclear leukocytes into CNS and peripheral tissue beds [1-5]. Elevated CNS expression of CCL2 has been a consistent observation among the different paradigms of experimental autoimmune encephalomyelitis (EAE) [6-8], a CNS demyelinating inflammatory disease that serves as a model for multiple sclerosis. This chemokine’s singular importance in driving EAE was demonstrated by global CCL2 knockout (*KO*) mice (CCL2^-/-^), which showed diminished severity and delay in onset of disease in C57BL/6 mice actively immunized with myelin oligodendrocyte glycoprotein_35-55_ (MOG_35-55_) [9]. Adoptive transfer EAE experiments also revealed effector T cells from MOG-immunized CCL2^-/-^ mice could transfer EAE to naïve wild-type (*WT*) recipients, while encephalitogenic T cells from *WT* donors were unable to induce EAE in CCL2^-/-^ mice [9]. Bone marrow chimera studies further showed active immunization EAE was markedly reduced when bone marrow from *WT* mice was engrafted into lethally irradiated CCL2^-/-^ mice, but not when bone marrow from CCL2^-/-^ mice was transferred into *WT* recipients [10]. Collectively, these findings of induced CNS expression of CCL2 during EAE, together with the adoptive transfer and bone marrow chimera studies, are consistent with a prominent role for CNS CCL2 in mediating EAE and diminish or negate the pathogenic impact of CCL2 from the peripheral leukocyte compartment.

What remains unclear, however, is which specific sources of CCL2 significantly contribute to disease, whether any reside locally in the CNS, their pathogenic mechanisms, and how they might be targeted therapeutically. Astrocytes are a major CNS source of CCL2 in both EAE and multiple sclerosis (MS) [7,8,11]. By projecting their endfeet toward the abluminal surface of brain microvascular endothelial cells (BMEC) that form the blood–brain barrier (BBB), astrocytes are ideally situated to intimately modulate BBB function and CNS leukocyte extravasation [12]. Our laboratory [13] and others [14,15] have demonstrated that CCL2 can disrupt integrity of cultured BMEC along with causing redistribution and reduction in expression of tight junction (TJ) proteins. *A priori*, CCL2 released from astrocyte endfeet may be partly responsible for the loss of BBB properties that accompanies both EAE [16] and MS [17], assisting development of a chemotactic gradient across the microvascular wall to drive the migration of adhered leukocytes past the endothelium, and/or further guiding extravasated leukocytes into the CNS parenchyma [18]. In addition to astrocytes, BMECs have also been shown to express CCL2 during EAE [19] and MS [20]. The observations that anti-CCL2 antibody prohibits firm attachment of leukocytes *in vivo* to CNS pial venules of mice immunized for EAE [21], and inhibits monocyte transendothelial migration across cultured BMEC [22], support the concept that CCL2 presented on the luminal endothelial surface aids in arresting leukocytes prior to their extravasation. The recent description that transendothelial migration of lymphocytes is mediated, in part, by intraendothelial vesicle stores of CCL2 [23], further accents a novel role for the endothelium as a critical source of this chemokine.

To resolve the respective contributions of astrocyte and endothelial cell CCL2 to neuroinflammation, we developed cell-conditional chemokine *KO* mice, in which the CCL2 gene was selectively eliminated in each of these cell types [24]. Here we report for the first time that targeted CCL2 gene deletion from either astrocytes or endothelial cells abates EAE pathogenesis, while differentially affecting separate aspects of CNS leukocyte extravasation and clinical disease course.

## Materials and methods

### Animals

Astrocyte and endothelial cell-specific CCL2 knockout (*KO*) mice were generated by intercrossing mice containing a floxed CCL2 allele with transgenic mice of glial fibrillary acid protein (GFAP)-Cre or Tie2-background, respectively, and have been previously characterized in detail [24]. Astrocyte-specific *KO* mice are referred to as *Astro KO*, and endothelial specific *KO* mice as *Endo KO* mice throughout this study. *KO* mice and their wild-type (*WT*) littermate controls were housed in specific pathogen-free conditions. All procedures involving animals were performed in accordance with the Animal Care and Use Guidelines of the University of Connecticut Health Center(Animal Welfare Assurance #A3471-01).

### EAE induction

EAE was induced by active immunization with MOG_35-55_ peptide (MEVGWYRSPFSRVVHLYRNGK; W. M. Keck Biotechnology Resource Center, Yale University), as previously described [25]. Briefly, on day 0 (d0), female mice 8 to 10 weeks of age were injected subcutaneously into the right and left flanks with a total of 300 μg of MOG peptide in complete Freund’s adjuvant containing 300 μg *Mycobacterium tuberculosis* (DIFCO). Mice were also injected intraperitoneally with 500 ng pertussis toxin (List Laboratories) in phosphate buffered saline (PBS, Gibco/BRL) on d0 and d2 post-immunization (p.i.) to heighten the autoimmune reaction to MOG_35-55_ peptide [26,27].

### Clinical assessment of EAE

Mice were scored daily for clinical disease severity according to the following scale: 0 = normal; 1 = tail limpness; 2 = limp tail and weakness of hind legs; 3 = limp tail and complete paralysis of hind legs; 4 = limp tail, complete hind leg and partial front leg paralysis; and 5 = death. Several disease parameters were calculated as described [28]. The Mean Day of Onset was calculated by averaging the time when clinical symptoms; i.e., a clinical score of ≥1, were first observed for two consecutive days in each mouse. The Mean Maximum Clinical Score was calculated by averaging the highest score for each mouse. The Disease Index was calculated by adding the daily average clinical scores in each group, dividing by the mean day of onset, and multiplying by 100. If an animal showed no disease, the day of onset was arbitrarily counted as one day after the last day of the experiment. Disease Incidence was the fraction of mice experiencing EAE.

### Cell culture and cytokine assay

MOG_35-55_-immunized mice were sacrificed and draining lymph nodes were dissected on d12. Mashed lymph nodes were pressed through a 70 μm mesh into cold RPMI. Cells were pelleted at 450 *g* at 4°C for 5 min and resuspended in red blood cell lysis buffer (Sigma) on ice for 5 min. After three washes with cold PBS, cells were stained with 0.4% Trypan blue (Sigma Aldrich) and counted with the Countess® Automated Cell Counter (Invitrogen) to permit discrimination of dead cells.

Single cell suspensions of lymph node cells (LNCs) were prepared and cultured in 24-well plates (Corning) at 1 × 10^6^ viable cells/mL in RPMI 1640 supplemented with 10% fetal bovine serum, 1.25% HEPES buffer, 1% sodium pyruvate, 1% penicillin-streptomycin, 1% glutamine, 1% non-essential amino acids, 0.01% 0.05M 2-mercaptoethanol (Sigma Aldrich). LNCs were restimulated with a combination of 10 μg/mL MOG_35-55_ and 0.5 ng/mL interleukin-12 (IL-12) (R&D Systems). Cytokines present in the cell culture supernatants of LNC were quantified using the multiplex enzyme-linked immunosorbent assay (ELISA) kit (R&D Systems).

### Proliferation assay

LNCs were prepared as for the cytokine assay. Cells were pulse-labeled with 2 μΜ carboxyfluoresceinsuccinimidyl ester (CFSE, Molecular Probes) in RPMI for 5 min at room temperature. After extensive washing with PBS, the CFSE-labeled cells were suspended and cultured in complete medium, as above, in 24-well plates (2 × 10^6^/well) for 72 h. Cell viability was assessed by Trypan blue exclusion. The LNC samples were washed in fluorescence-activated cell sorting (FACS) buffer (1% FCS and 0.1% sodium azide in PBS). After blocking with F_c_ Block (BD Biosciences) at 4°C for 20 min in the dark, the cells were washed and stained with fluorochrome-labeled antibodies against murine CD3, CD4, and CD11a (BD Biosciences) at 4°C for 30 min. After the cells were washed, the fluorescence intensities were measured by a FACS LSRII flow cytometer (BD Biosciences) and the following parameters were determined using FlowJo software (Treestar): % divided (percentage of cells in original population that has divided); Division Index (average number of cell divisions that a cell in the original population has undergone); Proliferation Index (total number of divisions**/**number of cells that went into division); Expansion Index (fold-expansion of overall culture); and Replication Index (fold-expansion of only responding cells).

### MOG_38-49_ MHC class II tetramer binding assay

The MOG_38-49_ MHC class II tetramer binding assay was based on that of Cravens et al. [29]. LNCs from MOG_35-55_-immunized mice were prepared and cultured as for cytokine and proliferation assays for 72 h. Thereafter, LNCs were washed with FACS buffer, and blocked with F_c_ Block in FACS buffer at 4°C for 20 min in the dark. LNCs were washed again, and MOG_38-49_ major histocompatibility complex (MHC) class II tetramer-PE or MHC class II control tetramer-PE hCLIP_103-117_-PE (obtained from the NIH Tetramer Core Facility) were added and incubated at 37°C for 1 hour in the dark. After incubation, the cells were directly stained with fluorochrome-labeled antibodies against murine CD4, CD11a, and CD44 at 4°C for 30 min. LNCs were washed and resuspended in FACS buffer. Samples were acquired on a FACS LSRII flow cytometer (BD Biosciences) and the data analyzed using FlowJo software (Treestar). Cells were gated as single live CD4^**+**^ T lymphocytes and examined for CD11a, CD44, and tetramer reactivity. Cells that were MOG tetramer-positive and CD11a^**+**^ were considered MOG-specific and previously activated.

### Immunofluorescence and 3D analysis of confocal z-stack images

Tissue was prepared as described by Paul et al. [30]. In brief, following transcardiac perfusion/fixation of mice, spinal cords were removed by laminectomy and freeze-embedded in cryomatrix. Subsequently, 12 × 60 μm cryosections from the thoraco-lumbar region, approximately between the T10 and L3 vertebrae were adhered to poly-L-lysine coated slides. Following staining, sections were mounted in Mowiol® prior to microscopic analysis.

For immunodetection of CCL2, affinity-purified rabbit anti-mouse CCL2 (Peprotech) was utilized with a corresponding anti-rabbit Alexa® 555-conjugated antibody (Life Technologies). Sections were subsequently double-immunostained with either rat anti-mouse CD31 (BD Pharmingen) followed by secondary incubation with anti-rat Alexa® 488 (Life Technologies) to highlight the endothelium, or Alexa® 488-conjugated anti-mouse GFAP (Life Technologies) to identify astrocytes. To specifically enhance detection of CCL2, which shows dispersed punctate immunoreactivity, confocal z-stacks were first deconvolved using AutoQuant ×3 (Media Cybernetics) software to correct for z-axis distortion. This significantly improved z-resolution and the resulting high-resolution images were exported into Imaris® (Bitplane Inc.). Representative z-slices, showing the co-localization of CCL2 with endothelial CD31 or astrocyte marker GFAP were then obtained.

For 3D quantification of microvascular tight-junction protein claudin-5 (CLN-5) density, the protocol recently detailed by Paul et al. [30] was used. The microvascular basement membrane (BM), a fusion of the respective endothelial and parenchymal BMs [31], was labeled with rabbit anti-mouse Laminin 1 (Lam 1) (Cedarlane) and anti-rabbit Alexa® 555 (Life Technologies). Anti-mouse Claudin-5-Alexa® 488 (Life Technologies) was employed to highlight TJs. Additionally, nuclear stain DRAQ5 (Biostatus Ltd.) was utilized to reveal the perivascular cellularity surrounding inflamed CNS microvessels, identified as venules [30], due to leukocyte extravasation.

Spinal cord venules from comparable regions of the dorsolateral white matter were imaged. Confocal z-stacks were acquired at 1-μm increments between z-slices, following a multitrack scan, using a Zeiss LSM 510 Meta confocal microscope equipped with a 40× Fluar (NA 1.3) and a 63× Plan-neofluar (NA 1.25) oil immersion lens. Confocal z-stacks were imported into Imaris® (version 7.6) software (Bitplane Inc.) and the venule of interest was segmented out from rest of the 3D dataset by manually tracing the vessel contour in each confocal z-slice, followed by merging the z-slice contours into a 3D contour surface. Surface area of the generated 3D contour was used as an estimate of the microvascular “surface area” defined by the endothelial layer. The CLN-5 channel was isosurface rendered (within the 3D contour surface) and the density of CLN-5 staining calculated as Total CLN-5 intensity*/*Microvascular surface area*.* CLN-5 density values were expressed as mean ± standard error of the mean (SEM).

To optically isolate (3D segmentation) and resolve the DRAQ5^+^ cellularity associated with leukocyte accumulation in the luminal or perivascular compartments of an inflamed venule [30], confocal z-stacks of venules revealing a cross-sectional view were acquired and imported into Imaris®. The venule of interest was then isolated from rest of the 3D dataset by creating a 3D contour surface, defining the parenchymal BM. This effectively eliminated the parenchymal cellularity (i.e., extravascular infiltrates). Additionally, the lumen was segmented out in a similar manner, by creating another 3D surface, approximating the contour of the endothelial BM (Endo BM). Spatial location of the observed cellularity between the BMs was considered perivascular (Additional file 1).

To graphically resolve the distribution of DRAQ5^+^ luminal and perivascular cells along microvascular x, y, and z axes with respect to the endothelial and parenchymal BMs, a 3D volume was first constructed from the acquired confocal z-stack, followed by 3D segmentation of the luminal and perivascular compartments as described above. Imaris® spot creation wizard was then employed to represent each of the DRAQ^+^ nuclei as a “spot object” in 3D space. Only DRAQ5^+^ nuclei >3 μm in diameter were considered. The 3D profiles of the created spot objects, representing the position of luminal and perivascular leukocytes, were then plotted on a 3D Imaris® Vantage plot to reveal its spatial location.

### Immuno-electron microscopy (Immuno-EM)

Colloidal gold detection of CNS microvascular CCL2 immunoreactivity by immuno-EM was performed as described previously [32]. *WT* mice at d16 EAE were anesthetized as described above, and subjected to transcardiac perfusion/fixation with Ringer’s solution, pH 6.9, followed by 4% paraformaldehyde, 0.1% glutaraldehyde in 0.1M phosphate buffer (PB), pH 7.4. Vibratome sections of spinal cord (300–500 μm thick) were cryoprotected with 2M sucrose in PB and plunge-frozen in liquid propane cooled by liquid nitrogen (-186°C). Sections were stained *en bloc* with 1.5% uranyl acetate in anhydrous methanol at -90°C for 30 hours and infiltrated with Lowicryl HM20 resin (Polysciences), followed by polymerization with UV light for 72 hours in a freeze-substitution instrument (Leica AFS) in a temperature gradient (-45°C to 0°C). Sections (70–80 nm thick) were cut and collected onto 400-mesh gold-gilded nickel grids coated with a Coat-Quick “G” pen (Daido). Tissue sections were incubated with anti-mouse CCL2 antibody (Peprotech), followed by incubation with goat anti-mouse IgG labeled with colloidal gold particles of 12 nm diameter (Jackson ImmunoResearch). After immunoreaction, tissue sections were counterstained with 2% uranyl acetate and then with 2% lead citrate. Primary antibody was omitted in immunoreaction as a control, and yielded no detectable gold labeling.

### Statistical analysis

For analysis of cell-specific *KO* on clinical EAE parameters, a χ^2^ test was used for comparisons of disease incidence; a Mann–Whitney U-test was used for comparisons of disease severity; and ANOVA, followed by Bonferroni’s multiple comparison post-hoc analysis, were used for comparison of disease onset [33]. ANOVA/Bonferroni post-hoc tests were also employed to assess differences in CLN-5 density values [30]. To contrast the rate of rise of clinical EAE progression among the different mouse groups, linear regression was performed on data points beginning at the onset of disease through attainment of the plateau or highest score. Statistical analyses were performed employing Prism 5 software (GraphPad), and results were considered significant at *P* ≤0.05.

## Results

### *Astro KO* and *Endo KO* mice show cell-selective loss of CCL2

**Figure 1:**
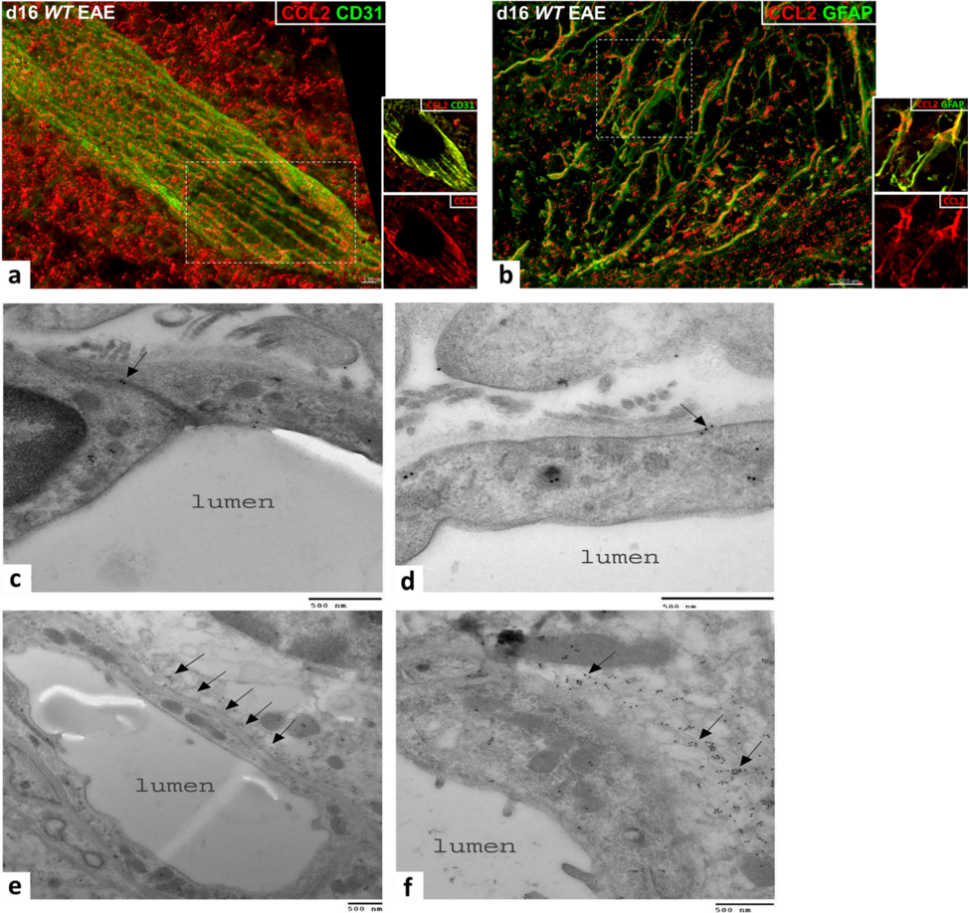
**CCL2 expression in spinal cord of *WT *mice during EAE. (a–b)** z-stack confocal images from spinal cord cryosections of *WT *mice at d16 EAE are shown, revealing staining of CCL2 **(red)**, and CD31 or GFAP **(green)** to delineate the endothelial cells and astrocytes, respectively. CCL2 staining was isosurface rendered for enhanced spatial perspective. **(a)***WT* mice express CCL2 both along the CD31^+^ microvascular endothelium, where staining appears aligned along the endothelial junctions, and within the perivascular space (left). **(b)** CCL2 staining is also associated with GFAP^**+**^ astrocytes (right). Insets show co-localization of CCL2 with CD31 or GFAP (yellow) in a single z-slice from the respective regions marked by the hatched white boxes, or CCL2 **(red)** channel alone. **(c–f)** Colloidal gold immuno-EM localization of CCL2 localization along microvessels in sections of spinal cord from mice at d16 EAE. **(c)** CCL2 immunoreactivity is localized within the inter-endothelial junction (arrow) and scattered throughout the endothelial cytoplasm. **(d)** A cluster of CCL2 immunoreactivity (arrow) is shown in close apposition to an endothelial vesicular structure that is near the plasma membrane. **(e)** Low magnification showing cross-section of a microvessel (possibly a postcapillary venule or small venule) and punctate distribution of CCL2 immunoreactivity in the perivascular space (arrows). **(f)** Higher magnification, revealing a high density of CCL2 immunoreactivity in and around what may represent astrocyte endfeet (arrows). Results are representative of 5–7 sections sampled from three mice in each group and two independent experiments. Scale bars are noted on the respective images.

Immunofluorescent staining of CCL2 in spinal cord during EAE in *Astro KO*, *Endo KO*, and *WT* mice is shown in high-resolution z-stack confocal images in Figure 1. Using identical image acquisition parameters, no CCL2 staining was detected either in naïve mice, or EAE mice in the absence of primary antibody (Additional file 2: Figure S1), thus highlighting specific immunoreactivity to inflamed CNS tissue. In *WT* mice at d16 post-EAE induction, CCL2 staining was vessel-associated as well as within the perivascular space (Figure 1a). Notably, CCL2 staining appeared aligned with inter-endothelial junctions (CD31), and showed a punctate distribution rather than a diffuse appearance throughout the cytoplasm. This could reflect containment of CCL2 within vesicles, as recently described by Shulman et al. [23] for cultured human umbilical vein endothelial cells (HUVECs). A representative z-slice further revealed intense double staining (CCL2 and CD31) of the endothelial layer. Parenchymal CCL2 staining (Figure 1b) was largely observed in association with GFAP^**+**^ astrocytes. Localization within astrocytes was confirmed in a representative z-slice. Some large deposits of CCL2 could also be seen just outside the astrocytes, which may indicate secreted chemokine. Both endothelial and astrocyte immunoreactivity in *WT* mice with EAE were confirmed by immuno-electron microscopy (Figure 1c–f). Notably, CCL2 immunoreactivity was detected within the inter-endothelial junctions (Figure 1c), and could also be seen in association with endothelial vesicular-like structures (Figure 1d). There was also abundant immunoreactivity within cellular processes at the abluminal side of the endothelium, possibly representing CCL2 destined for or contained within astrocyte endfeet (Figure 1e–f).

**Figure 2:**
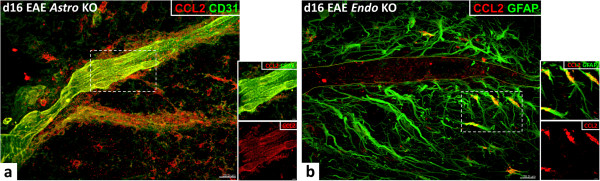
**CCL2 expression in spinal cord of *Astro KO *and *Endo KO *during EAE.** Representative z-stack confocal images from spinal cord cryosections of *KO* mice at d16 EAE are depicted. Cell-specific CCL2-*KO* mice display loss of CCL2 staining in respective targeted cell types. **(a) ***Astro KO* mice show venule-associated CCL2 staining, but lack staining in the parenchymal astrocytes (left). **(b)** In contrast, *Endo KO* mice are deficient in vessel-associated CCL2 staining, but maintain astrocyte staining (right). The endothelial boundary is marked with **yellow lines**. Insets show co-localization of CCL2 with CD31 or GFAP **(yellow)** in a single z-slice from the respective regions marked by the hatched white boxes, or CCL2 **(red)** channel alone. Results are representative of 5–7 sections sampled from three mice in each group and two independent experiments.

The CCL2 staining patterns with both *KO* mice were markedly different (Figure 2). *Astro KO* mice at d16 (Figure 2a) showed vessel-associated CCL2 staining but greatly diminished staining in the perivascular space. Conversely, *Endo KO* mice (Figure 2b) exhibited a near absence of vessel-associated CCL2 staining, while robust CCL2^**+**^ astrocytes were still clearly evident. These staining patterns are consistent with previous results from this laboratory showing CCL2 RNA expression by both microvessel and parenchymal fractions of brain and spinal cord from *WT* mice with EAE [33], and argue that CCL2 protein is produced by astrocytes and BMECs, and not merely taken up at these sites.

That some vessels in *Astro KO* mice and some astrocytes in *Endo KO* mice appeared devoid of CCL2 staining possibly reflects that not all of the respective endothelial cell and astrocyte populations became similarly inflamed, a prospect supported by only a subset of vessels/vessel segments being associated with leukocyte infiltrates (data not shown). It may further be that CCL2 from endothelial cells or astrocytes exerts some positive control over CCL2 expression by the other cell type [24,34].

### *Astro KO* and *Endo KO* mice are both resistant to EAE, but show different clinical phenotypes

**Figure 3:**
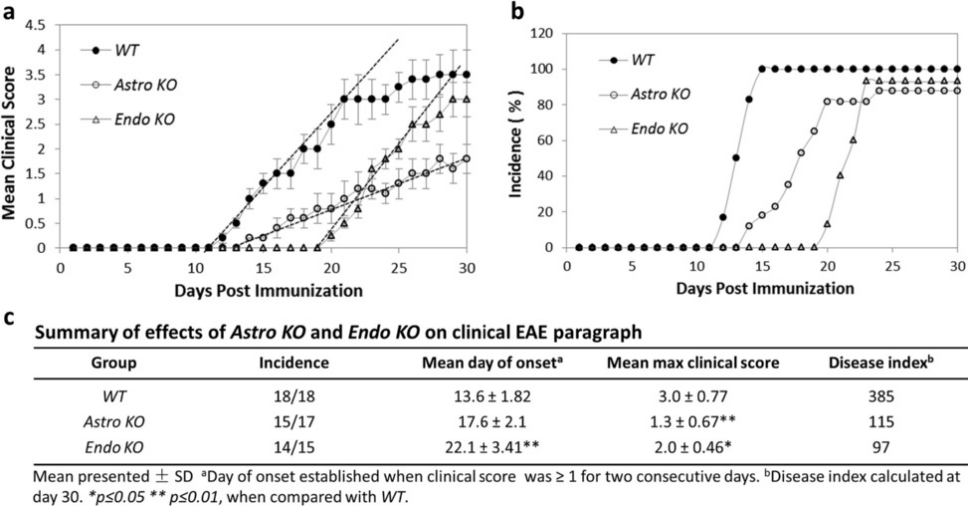
***Astro KO *and *Endo KO *mice show different patterns of clinical EAE.** EAE was induced in *WT*, *Astro KO*, and *Endo KO* mice by MOG_35-55_ immunization; all mice were observed daily and scored for clinical disease for 30 days. Each group consisted of 5–6 mice, and analysis was performed in triplicate. Graphs represent mean data points from all three analyses ± standard error. **(a)** Mean clinical EAE scores. *Astro KO* mice do not attain as severe disease as *WT* during the evaluation period, while *Endo KO* mice approach *WT* disease severity but do so only after significantly delayed onset. The rate of rise of clinical disease, as reflected by the slope of each regression line (hatched lines) through the respective ascending disease scores for the different mice, is similar in both *WT* and *Endo KO* mice, but notably less in *Astro KO* mice. **(b)** Disease incidence. All mice show a similar incidence of disease but, compared to *WT* mice, *Astro KO* mice show only a mild delay while *Endo KO* mice show a prolonged delay in disease onset. **(c)** Summary of various clinical disease parameters among the three mouse groups.

Figure 3a,b shows that targeted CCL2 gene eliminations in astrocytes and endothelial cells, respectively, prominently affected development of EAE, consistent with earlier reports of the effects of global CCL2 knockout [9,10]. While the incidence of disease was largely unaffected in both *KO* mice compared to *WT*, there was clear evidence of altered disease progression in the two cell-selective CCL2 *KO* groups*. Astro KO* mice exhibited significantly reduced disease severity (clinical score) compared with *WT* littermates throughout the time frame observed, along with just a slight, insignificant delay in disease onset. In sharp contrast and nearly mirror image effect, *Endo KO* mice showed only mild reduction in disease severity, but a significantly protracted delay in the onset of disease. It was further observed that the rate of rise of clinical disease, as reflected by the slope of the linear, ascending region of the disease score graphs, was different between *Astro KO* and *Endo KO* mice. *Astro KO* mice showed a lesser rate of rise of clinical disease than both *WT* and *Endo KO* mice, while the latter two mouse groups showed similar rates of disease rise. Thus, it appears that while disease was significantly delayed in *Endo KO* mice, once disease commenced it proceeded on a normal time course (for the period evaluated). Comparisons of Disease Incidence, Mean Day of Onset, Mean Maximum Clinical Score, and Disease Index in *Astro KO*, *Endo KO*, and *WT* mice are tabulated in Figure 3c.

### *Astro KO* and *Endo KO* mice do not show different MOG-specific T cell responses

**Figure 4:**
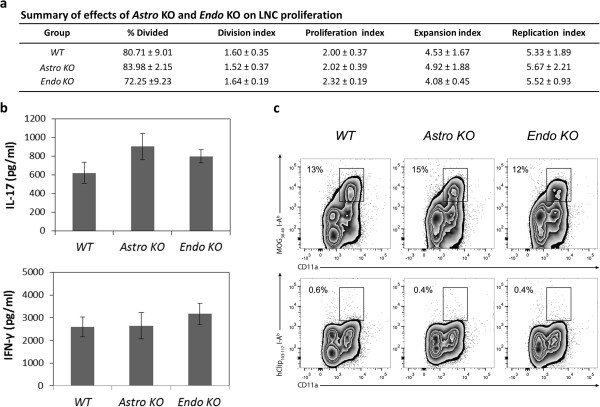
**LNCs from MOG**_**35-55**_**-immunized *WT, Astro KO*, and *Endo KO *mice show similar responses to MOG**_**35-55**_**restimulation *in vitro*.** LNCs were prepared from MOG_35-55_-immunized mice on d12, and restimulated with MOG_35-55_ for 72 h in culture, after which time different responses were measured. **(a)** T cell proliferation. LNC were pulse-labeled with 2 μΜ CFSE for 5 min at the beginning of culture and analyzed after 72 h by FACS, gating on CD3, CD4, CD11a. **(b)** Cytokine production. The concentrations of IL-17 and IFN-γ were determined in supernatants of LNC after 72 h in culture. **(c)** Binding of MOG_38-49_ MHC class II tetramer-PE. Binding was determined after 72 h in culture, and hCLIP_103-117_ tetramer-PE served as a control for non-specific binding. Plots were gated on CD4^+^ T lymphocytes. The frequency of MOG_38-49_ I-A^b^ tetramer^+^ CD4^+^ T cells is similar among *WT*, *Astro KO*, and *Endo KO* groups, while hCLIP_103-117_ I-A^b^ tetramer does not bind cultured T cells. The data shown are representative of at least two independent experiments; data in **(a)** and **(b)** reflect mean value ± standard error.

*Endo KO* mice have the CCL2 gene eliminated from all endothelial cells, central and peripheral. This could mean that observed alterations in EAE disease progression in *Endo KO* mice might stem from a defect in the afferent immune arm, such as attenuated T cell priming. Indeed, this possibility is prompted by findings that CCL2 is expressed and presented by high endothelial venules in lymph nodes [35], instrumental in dendritic cell maturation [36], and released by dendritic cells to attract antigen-specific T cells [37]. Hence, several T cell proliferation parameters – % Divided, Division Index, Proliferation Index, Expansion Index, and Replication Index *–* were assayed in CSFE pulse-labeled LNC cultures from MOG-immunized *Astro KO*, *Endo KO*, and *WT* mice (Figure 4a). Following restimulation with MOG, no significant differences were detected among cells from the three types of mice for any of these parameters. This is in agreement with previous findings that indicated no difference in T cell proliferation between LNC cultures from *WT* and global CCL2^-/-^ mice [9].

We also assayed the ability of cultured LNCs from each of these mice to produce IFN-γ or IL-17, which are cytokines considered instrumental in autoimmunity and EAE [38], in response to restimulation with MOG. Figure 4b shows that MOG-stimulated expression of neither cytokine was significantly altered in *Astro KO* or *Endo KO* mice compared to their *WT* cohorts. Earlier, Huang et al. [9] had described diminished production of IFN-γ in LNCs from CCL2^-/-^ mice, which might reflect the effects of having depleted a peripheral CCL2 pool distinct from that of endothelial cells. Figure 4c further reveals that, following MOG immunization, the percentage of MOG_38-49_ MHC class II tetramer positive CD4 T cells was in line with another report [29] and similar in LNCs from *Astro KO*, *Endo KO*, or *WT* mice, indicating that the frequency of MOG-specific T cells was comparable among these groups. Hence, our results argue against either astrocyte- or endothelial cell-targeted CCL2 gene deletion having significantly impaired peripheral T cell behavior and immune responsiveness to MOG peptide. These findings are further in accord with reports that MOG-specific, encephalitogenic T cells can be generated in mice with global knockout of CCL2 [9].

### *Astro KO* and *Endo KO* mice display altered inflammatory responses along the CNS microvasculature during EAE

**Figure 5:**
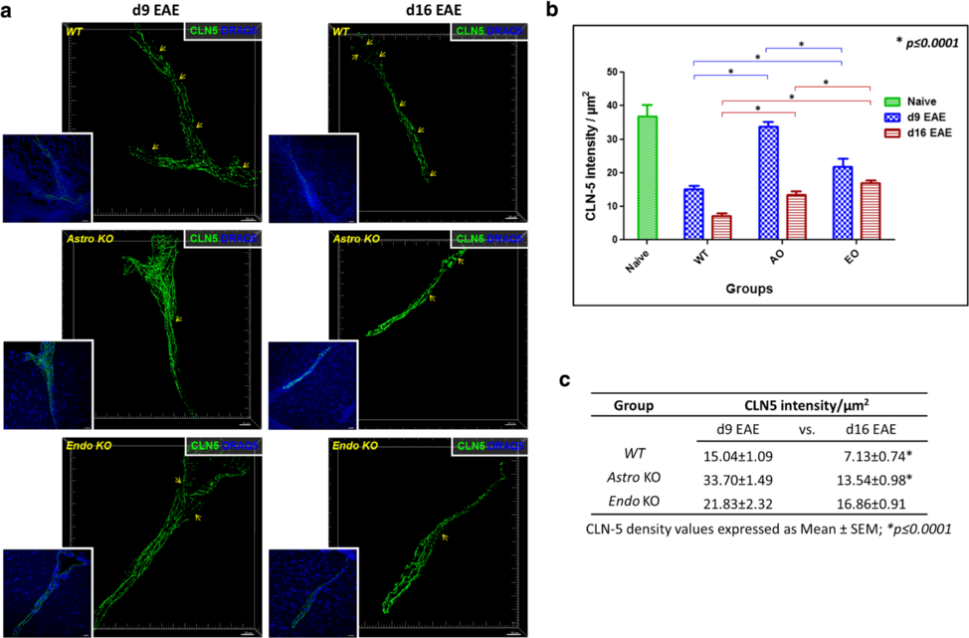
***Astro KO *and *Endo KO *mice show differential loss of CLN-5 staining in spinal venules during EAE. (a)** Isosurface-rendered images were generated from confocal z-stacks of 60 μm thick thoraco-lumbar spinal cord cryosections at d9 and d16 EAE, as described in Materials and Methods. Staining of CLN-5 **(green isosurface)** and nuclei/DRAQ5 **(blue)** is shown. Larger images displaying 3D perspective projection views of confocal reconstructions show CLN-5 channel only, to emphasize the fragmented pattern of TJ protein staining. Inserts depict both CLN-5 and nuclei, highlighting the close association of altered CLN-5 staining with dense perivascular cellularity representing infiltrating leukocytes. Arrows demark overt gaps in CLN-5 staining, where the TJ pattern is clearly disrupted. Notably, CLN-5 staining pattern during EAE appears most intact in *Astro KO* mice, least so in *WT* mice, and intermediate in *Endo KO* mice. **(b)** Quantification of CLN-5 staining as intensity per unit surface area of the endothelium. CLN-5 density in naïve *WT* mice is included as a reference for the normal state, wherein the pattern of CLN-5 junctional staining is continuous [30]. Statistical comparisons are between groups and within days. **(c)** Summary of CLN-5 changes. Statistical comparisons are within groups and between days. A total of 12 venules were analyzed in each group sampled from three mice. Data reflect mean value ± standard error. Scale = 20 μm.

Given the different clinical EAE phenotypes of *Astro KO* and *Endo KO* mice with apparent absence of overt impact on T cell priming, we next sought to determine effects of cell selective CCL2 loss on inflammatory events along the CNS microvasculature. 3D perspective projection views of confocal reconstructions were generated using Imaris® to provide a more realistic 3D representation of the z-stack dataset. Notable differences in staining of CLN-5, a prominent TJ protein in CNS microvessels and BBB determinant [39], were found among *WT*, *Astro KO*, and *Endo KO* mice during EAE (Figure 5), while naïve mice of all groups showed no evidence of disparities (data not shown). At d9, prior to evidence of clinical disease, *WT* mice showed focal fragmentation of the CLN-5 staining pattern. This discontinuous appearance of CLN-5 staining in regions of increased perivascular cellularity was not a result of CLN-5 staining being distributed in z-planes not captured during acquisition, as the confocal reconstructions shown represent 3D images generated from 60 × 1-μm thick z-slices. Rather, the areas lacking immunostaining reflected actual sites of CLN-5 disruption associated with extravasating leukocytes. This picture differed significantly from that seen with naïve mice, where CLN-5 immunostaining appeared continuous, without obvious interruptions, and perivascular cellularity was absent (Additional file 3: Figure S2). Hence, for the purpose of highlighting the pathologic role(s) of CCL2 released from astrocyte and endothelial sources in neuroinflammation, statistical comparisons for CLN-5 density were only made between *WT*, *Astro KO*, and *Endo KO* mice during evolving EAE. The significantly altered CLN-5 pattern observed in *WT* mice during EAE corresponded with a sharp decrease of approximately 60% in the density of CLN-5 staining. In stark comparison, *Astro KO* mice demonstrated little if any change in CLN-5 staining, while *Endo KO* mice displayed a somewhat intermediate response at this time-point. By d16- following disease onset- *WT* mice showed further disruption and loss of CLN-5 staining, down approximately 80% from that demonstrated by naïve mice. *Astro KO* mice at this later time showed a precipitous loss of CLN-5 staining, declining by approximately 60% (compared to naïve and d9 mice), while *Endo KO* mice at d16 showed only a moderate, non-significant CLN-5 decrease of approximately 19% (compared to d9).

**Figure 6:**
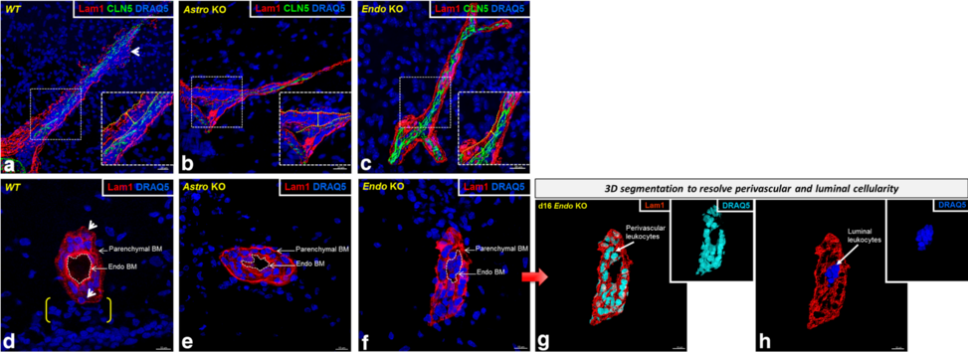
***Astro KO and Endo KO *mice display differences in perivascular cellularity associated with spinal venules durin*g *EAE.** Isosurface-rendered images were generated from confocal z-stacks of 60 μm cryosections at d16 EAE. Staining of BM Lam 1 **(red)**, CLN-5 **(green)**, and nuclei/DRAQ5 **(blue)** is shown. **(a, b, c)** Longitudinal sections reveal the extent of vessel-associated leukocytes. CLN-5 staining is presented to highlight the endothelial boundary. Insets represent enlarged view of areas highlighted in white hatched boxes, while double-headed arrows denote the space between the endothelial and parenchymal BMs. All extravasated leukocytes within this space are considered “perivascular”. In *WT* mice, a dense accumulation of DRAQ5^+^ perivascular cells (representing leukocytes) is seen, a few apparently penetrating the fragmented parenchymal BM (arrowhead). In *Astro KO* mice, a similar dense perivascular cellularity is observed, with visibly intact parenchymal BM and lack of parenchymal leukocyte migration. In *Endo KO* mice, the BM is also apparently intact, with minimal perivascular cellularity. Scale = 20 μm. **(d, e, f)** Cross-sections highlight the spatial distribution of vessel-associated leukocytes. In *WT* mice, the vessel lumen (demarked by **white dashes**) appears empty and cells are seen in the perivascular space. A few cells are visibly penetrating the parenchymal BM (arrowheads), alongwith dense parenchymal cellularity (brackets). In *Astro KO* mice, the lumen again appears empty; congregated cells are evident in the perivascular space, with a few parenchymal clusters. In *Endo KO* mice, cells are clearly present in the lumen, with apparently fewer cells in the perivascular space as compared to *WT* and *Astro KO* mice. Parenchymal clustering is seemingly absent. The diffuse DRAQ5^+^ cells are likely parenchymal neural cells. **(g-h)** The **red arrow** designates the same *Endo KO* image subject to *contour-based 3D segmentation* (see Materials and Methods) to further resolve luminal (**blue**) from perivascular (**turquoise**) cells. Results are representative of 5–6 microvessels sampled from three mice in each group and two independent experiments. Scale = 10 μm.

**Figure 7:**
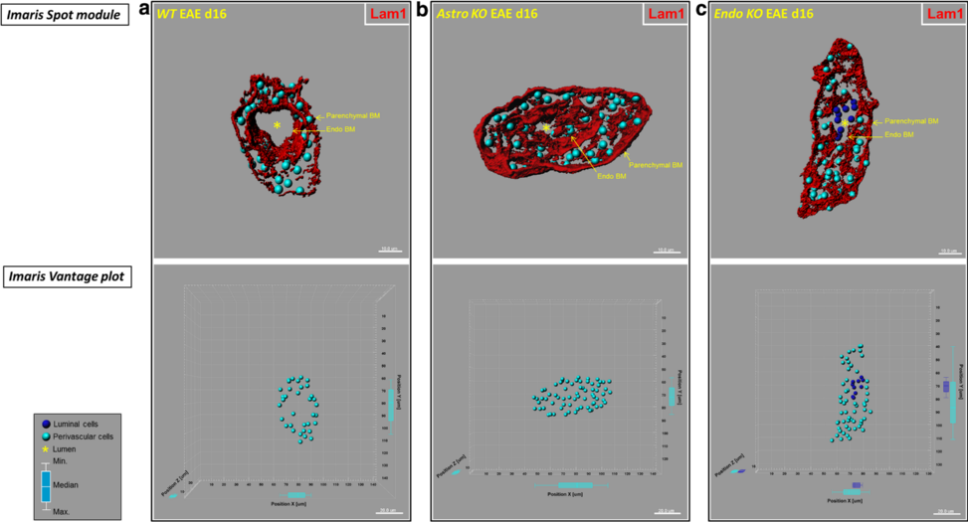
***Astro KO *and *Endo KO *mice display differences in 3D distribution profiles of luminal and perivascular cells.** Isosurface-rendered images were generated from confocal z-stacks of 60-μm thick cryosections from *WT*, *Astro KO*, and *Endo KO* mice at d16 EAE. The BM is highlighted by staining of Lam1 **(red). (Top row)** DRAQ5^+^ nuclei in luminal and perivascular compartments were optically isolated using 3D contour based segmentation (as described in Materials and Methods), and pseudo-colored **blue** (luminal) and **turquoise** (perivascular), respectively. Using Imaris® spot creation module, each of these nuclei is shown in the 3D dataset (volume) as a “spot object,” designating its luminal or perivascular location. Scale = 10 μm. **(Bottom row)** Imaris® vantage plots showing the 3D distributions of luminal and perivascular cells along microvascular x, y, and z-axes in the corresponding vessels from the top row. Scale = 20 μm. **(a)** Representative *WT* vessel showing an empty lumen (*). **(b)** The lumen in the *Astro KO* vessel also appears empty (*) but partially collapsed, possibly owing to accumulation of perivascular cells that are missing guidance cues from deleted astrocyte-derived CCL2. **(c)** In contrast, *Endo KO* vessel shows evidence of congregation of cells in the lumen **(blue)**, possibly reflecting stalled leukocyte transmigration in absence of endothelial-derived CCL2. Box-and-whisker plots are shown indicating the maximum and minimum spread from the median, in μm, of luminal or perivascular nuclei along the x, y, and z-axes.

Differences in leukocyte infiltration patterns were also obvious among the various strains of mice when vessels were viewed in longitudinal- and cross-section (Figure 6). During neuroinflammation, the BM splits into its respective endothelial and parenchymal components [31], highlighting two spaces: the subendothelial space (between the endothelium and endothelial BM) and the perivascular space (between the endothelial BM and the parenchymal BM). Both these spaces swell with leukocytes that have recently extravasated across the BMECs, but as the BM becomes extensively fragmented during this process, demarcation between the spaces is blurred in longitudinal sections. Thus, in referring to leukocyte distribution, the term “perivascular” is used herein to describe all extravasated leukocytes that are vessel-associated. At d16, dense perivascular infiltrates were seen to be associated with venules in *WT* mice, with cells apparently penetrating the parenchymal BM to enter the parenchyma (Figure 6a,d). *Astro KO* mice showed similar type clusters of perivascular cells, but no clear evidence of leukocytes in the act of rupturing the parenchymal BM (Figure 6b,e). Venules of both *WT* (Figure 6d) and *Astro KO* (Figure 6e) further exhibited lumens within which no cells were detectable. By contrast, *Endo KO* mice alone displayed aggregated cells apparently stalled in the lumen, as well as a seemingly lesser extent of perivascular cells (Figure 6c,f–h). To further resolve the aggregated cells throughout the microvascular lumen of an entire 60 -μm section from *Endo KO* mice, and graphically distinguish this pattern from that in *WT* and *Astro KO* mice, the distribution profiles of cells associated with the lumen and perivascular space, respectively, were mapped in 3D (Figure 7). Intra-luminal cells could only be detected in *Endo KO* mice (Figure 7c). As the actual number of spinal vessels showing any evidence of inflammation was extremely low in Endo *KO* mice at this time, the few examples detected showed this common appearance of hindered leukocyte migration. Moreover, since tissue was perfusion-fixed, these luminal cells are not likely to have resulted from blood stasis but, instead, suggest a possible deficit in extravasation from CNS microvessels in *Endo KO* mice.

## Discussion

**Figure 8:**
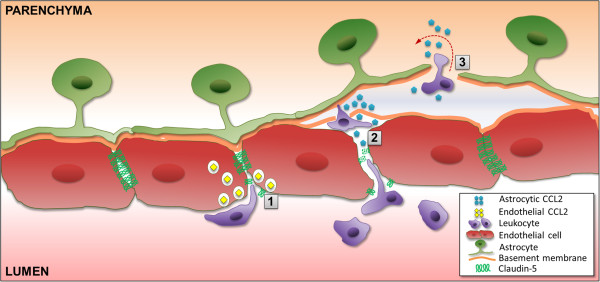
**Differential actions of astrocyte-derived and endothelial cell-derived CCL2 at CNS venules.** Based on observations with *Astro KO* and *Endo KO* mice during EAE, the schematic depicts endothelial-derived CCL2 facilitating migration across the endothelium (1), a step post-adhesion. Astrocyte-derived CCL2 is shown promoting both breakdown of endothelial tight junctions (2), and penetration of leukocytes across the parenchymal BM into the CNS parenchyma (3).

While the critical role of CCL2 in EAE has been revealed in global CCL2 knockout studies [9], and the chemokine sources mediating this effect are suggested to reside in the CNS [10], the identities of the sources responsible for specific neuroinflammatory events, e.g., effects at the BBB and leukocyte penetration into the CNS, have not been resolved. In the present study, we used mice with targeted CCL2 gene deletion in astrocytes or endothelial cells, along with 3D confocal imaging, to establish - for the first time – that CCL2 from each of these sources regulates different aspects of neuroinflammation and EAE course. *Astro KO* mice exhibited a similar onset but reduced severity of disease compared to *WT*, while *Endo KO* mice displayed nearly the opposite clinical pattern. However, neither of these *KO* mice showed any significant changes in MOG-specific T cell responses in LNC cultures, consistent with the observed effects of CCL2 gene deletion being limited to the CNS. Further reflecting CNS action, *Astro KO* mice failed to show the parenchymal leukocyte infiltration and clear disruption of CLN-5 that accompanied *WT* EAE, while *Endo KO* mice revealed leukocytes apparently stalled in the lumen of spinal cord microvessels. Significantly, the combined effect of separate astrocyte and endothelial CCL2 elimination on clinical disease closely mirrors the phenotype reported when CCL2 gene ablation was confined to the “central compartment” by adoptive transfer of *WT* T cells [9] or transplantation of *WT* bone marrow [10] into global CCL2 *KO* mice; i.e., reduced disease severity along with delayed disease onset. This reinforces the notion that CCL2 from astrocytes and endothelial cells each contribute to EAE disease in a major, yet different way. The differences in EAE noted between *Astro KO* and *Endo KO* mice may reflect direct consequences of cell-specific CCL2 release on the CNS microvascular endothelium, leukocyte migration, or both. Possible actions of CCL2 released by CNS endothelial cells or astrocytes are schematized in Figure 8.

### Astrocyte-derived CCL2 regulates BBB integrity and leukocyte penetration into the CNS parenchyma, while endothelial-derived CCL2 impacts leukocyte transendothelial migration

The failure of only *Astro KO* mice to show clear disruption of CLN-5 staining at d9 EAE supports the interpretation that CCL2 released from astrocytes figures prominently in destabilizing endothelial TJs at the abluminal microvascular surface early during the neuroinflammatory process [13]. Later loss of CLN-5 in these mice by d16 may, instead, reflect disruption of TJs by extravasating leukocytes. Astrocyte-derived CCL2 may additionally serve to recruit extravasated leukocytes into the parenchyma, as *Astro KO* mice revealed leukocytes congregated in the perivascular space. This latter role is supported by Carrillo-de Sauvage et al. [18], who described contact of CNS infiltrating T cells with CCL2-expressing perivascular astrocytes during neuroinflammatory disease.

The disruption of CLN-5-containing TJs during EAE and its prominent control by astrocyte-derived CCL2, is consistent with numerous reports on the action of CCL2 on TJs and BBB properties in cultured BMECs [13-15,40], CNS microvessels *in vitro*[13], and CNS microvessels *in vivo* in other neurological settings [41-43]. Notably, however, a recent report describing the effect of pertussis toxin injection in mice constitutively overexpressing CCL2 selectively in oligodendrocytes under direction of the myelin basic protein (MBP) promoter, found no evidence of a disrupted CLN-5 pattern accompanying leukocyte extravasation into brain [44]. This apparent contradiction may be due, in part, to the high level of chronic over-expression of CCL2 (<100,000 times normal values) having caused down-modulation of CCR2, the cognate receptor for CCL2, on BMECs [45], as well as inappropriate or inadequate access of oligodendrocyte-derived CCL2 – normally not found in health or disease – to the CNS microvasculature. The present study thus underscores the unique relationship between endogenous, astrocyte-derived CCL2, TJs, and BBB permeability in neuroinflammatory disease [46].

Endothelial-derived CCL2, on the other hand, may facilitate a ‘post-adhesion’ stage of leukocyte extravasation, as the absence of this chemokine pool was uniquely associated with the appearance of leukocytes stalled within the microvascular lumen. In support of this hypothesis, Shulman et al. [23] recently showed that an intraendothelial vesicle pool of CCL2 within cultured HUVECs is a critical regulator of transendothelial migration of adherent effector T cells. Our data, showing both apparently stalled leukocytes in the microvascular lumen of *Endo KO* mice and punctate CCL2 immunostaining in BMECs of *WT* mice, may thus represent an extension of the results of Shulman et al. [23] to an *in vivo* scenario and advance a critical role for a CNS endothelial pool of CCL2, possibly vesicle bound, in mediating leukocyte transendothelial migration during neuroinflammatory disease. While this differs from the finding that CCL2 facilitates adhesion of leukocytes to pial microvessels [21], this distinction may represent the considerable endothelial heterogeneity that exists along the CNS microvasculature [47].

The smaller but significant loss of CLN-5 staining noted in *Endo KO* mice from d9 to d16 may chiefly represent the action of astrocyte-derived CCL2, as lesser extravasation was observed in these mice during this period. Conceivably, the astrocyte CCL2 pool could also have guided the lesser amount of extravasated cells into the parenchyma, resulting in the delayed disease noted.

### Cell-selective CCL2 *knockout* highlights CNS actions of CCL2 in neuroinflammatory disease

Our collective findings reinforce critical and non-redundant roles of CNS CCL2 in mediating EAE, as previously implicated in adoptive transfer and bone marrow chimera EAE studies with global CCL2 knockout mice [9,10]. Of further importance, the use of *Astro KO* and *Endo KO* mice together with high-resolution 3D confocal imaging in this study was able to resolve apparently unique contributions of astrocyte and endothelial CCL2 pools to EAE pathogenesis. That both types of mice might share some effects of conditional CCL2 deletion is in accord with reports that BMECs can deposit CCL2 abluminally [48], and CCL2 can be transcytosed from the abluminal to luminal BMEC surface [34]. Because all endothelial cells in the *Endo KO* mice are deficient in CCL2 expression, at this time it cannot be concluded that CCL2 from CNS endothelial cells, as opposed to peripheral endothelial cells, affected the disease process. However, given the failure of LNCs from these mice to show any deficits in MOG-stimulated proliferation, IFN-γ or IL-17 production, or MOG MHC class II tetramer staining, it is doubtful that the absence of CCL2 from peripheral endothelial cells was a major factor in the aberrant EAE patterns noted. Diminished CLN-5 disruption and heightened presence of luminal leukocytes in *Endo KO* mice further point to the CNS endothelial pool of CCL2 as featuring critically in EAE.

Though only EAE was analyzed in this study, astrocytes and BMECs have been suspected as critical sources of CCL2 during other neuroinflammatory conditions investigating three different CNS inflammatory scenarios (human glioma, striatal injection of LPS in mice, and adenovirally injected monkeys) reporting that extravasation of lymphocytes is mediated by CCL2-expressing astrocytes independent of the inflammatory situation and species [18]. Further, Tei et al. [49] most recently argued that CCL2 expression by both astrocytes and BMECs may contribute to the invasion and parenchymal migration of brain Iba1^+^/NG2^+^ cells, descendants of subpopulations of circulating monocytes, following cerebral ischemia. Thus, expression of CCL2 by astrocytes and/or BMECs may be considered a widespread phenomenon associated with neuroinflammation. The seminal importance attributed to these particular sources of CCL2 does not preclude contributions by other CCL2-expressing cell types, e.g., microglia [10,50], which may further modulate neuroinflammatory disease in their unique ways.

It has nevertheless been firmly established through elegant adoptive T cell transfer [9] and bone marrow chimera [10] studies, that CCL2 derived from the peripheral leukocyte compartment is not critical to the development of EAE. Hence, even though Tie-2-driven Cre expression has been reported in cells of the hematopoietic lineage [51], any potential loss of CCL2 from this population would not detract from our interpretation that CCL2 elimination from BMEC, rather than leukocytes, predominantly altered EAE disease. The delay of disease phenotype in *Endo KO* mice, in fact, is consistent with the lack of CCL2 immunostaining in BMEC and apparent stalling of adherent leukocytes observed in these animals.

### Therapeutic targeting of CNS CCL2

Lastly, as the BBB has generally been recognized as the major impediment to drug delivery to the CNS [52], these results have significant implications for targeting CCL2 in the treatment of neuroinflammatory disease. It is thus notable to point out that injection of a CCL2-neutralizing antibody directly into the brain was effective at suppressing lymphocyte infiltration following striatal lipopolysaccharide injection [18], a situation in which astrocytes were observed to be the major CCL2-expressing cell type. Arguably, therapeutic inhibition of endothelial CCL2 would not require circumventing or penetrating the BBB, in contrast to suppressing astrocyte production of this chemokine. However, as our and the recent results of Shulman et al. [23] point out, merely targeting CCL2 with antibodies or receptor antagonists may not be effective against vesicle-bound endothelial CCL2 depots. The recent demonstration that the anti-inflammatory compound bindarit, a synthetic indazolic derivative (MWr 324 Daltons) that preferentially inhibits transcription of the monocyte chemoattractant subfamily of CC chemokines (MCP-1/CCL2, MCP-2/CCL8, and MCP-3/CCL7), delayed and suppressed EAE in concert with diminishing CNS microvascular CCL2 expression [33], suggests that interfering with intra-endothelial CCL2 might be of high therapeutic value. However, as BBB disruption often accompanies neuroinflammatory disease [30,47], drugs that inhibit CCL2 synthesis could potentially have opportunity to strike at both endothelial cells and astrocytes, even if only with limited efficiency at the latter, and thus offer better therapeutic prospects than antibodies or antagonists. Recent descriptions of CCL2 involvement in post-ischemic disruption of the BBB [43], beta-amyloid neurotoxicity [53], and traumatic brain injury [54], further underscore that modulating CNS CCL2 synthesis at the vascular and/or parenchymal level may offer a novel therapeutic option for a wide range of neuropathologies.

## Conclusions

In light of our results, it is determined that CCL2 from either astrocytes or BMECs separately impacts clinical EAE and associated neuroinflammatory processes in distinct ways and through different mechanisms depending on the source cell type. CCL2 from astrocytes regulates severity of clinical EAE disease, while controlling penetration of leukocytes into the CNS parenchyma and disrupting CLN-5 staining pattern along the CNS microvasculature. In contrast, CCL2 from BMECs appears to more so determine disease onset, and effect post-adhesion leukocyte transendothelial migration. Therapeutic targeting of CCL2 expression or action at the parenchymal and/or vascular levels may thus offer promise in treating neuroinflammatory disease.

## Abbreviations

Astro KO: Astrocyte-specific CCL2 knockout mice; BBB: Blood–brain barrier; BMEC: Brain microvascular endothelial cells; BM: Basement membrane; CCL2: Chemokine (C-C motif) ligand 2; CFSE: Carboxyfluorescein succinimidyl ester; CLN-5: Claudin-5; CNS: Central nervous system; EAE: Experimental autoimmune encephalomyelitis; Endo KO: Endothelial-specific CCL2 knockout mice; FACS: Fluorescence-activated cell sorting; GFAP: Glial fibrillary acid protein; HUVECs: Human umbilical vein endothelial cells; IL: Interleukin; Immuno-EM: Immuno-electron microscopy; Lam 1: Laminin 1; LNC: Lymph node cells; MHC: Major histocompatibility complex; MOG: Myelin oligodendrocyte glycoprotein; MS: Multiple sclerosis; PB: Phosphate buffer; PBS: Phosphate buffered saline; TJ: Tight junction; WT: Wild type mice.

## Competing interests

The authors declare that they have no competing interests.

## Authors’ contributions

JSP, SG, and DP designed the experiments and wrote the paper. DP, SG, and YL performed the experiments. ERJ assisted with LNC proliferation and FACS analysis. DRS acquired the EM images and NHR provided instruction with the EAE model. All authors read and approved the final manuscript.

## Supplementary Material

Additional file 1 **Contour-based 3D segmentation of luminal- and perivascular-associated cells in *Endo KO *** **mice during EAE.** The video shows 3D reconstruction of the representative *Endo KO* venule from Figure 7, allowing for enhanced visualization of the perivascular and luminal cellularity associated with the microvessel along x, y, and z axes. It further demonstrates the sequential steps employed for the contour-based 3D segmentation of DRAQ5^+^ cells into separate luminal and perivascular compartments, using 3D contour surfaces, as described in Materials and Methods. The nuclei were pseudo-colored in **blue** (luminal) and **turquoise** (perivascular). Each 180°-turn in the video indicates the following steps in sequence - 3D reconstruction **→** Lam1 (**Red**) isosurface **→** parenchymal BM contour → endothelial BM contour **→** segmented nuclei. Click here for file

Additional file 2: Figure S1 Specificity of CCL2 immunostaining. Volume rendered images of z-stacks obtained from serialsections of d9 EAE spinal cords used in Figure 6 (**left**) and naïve (**right**) mice demonstrating specific immunoreactivity of the CCL2 antibody. No detectable CCL2 staining (**green**) was observed in naïve mice upon incubation with CCL2 antibody or in EAE mice in the absence of primary antibody. The endothelium is highlighted with CD31 (**red**), while DRAQ5 staining reveals the nuclei (**blue**). Scale = 20 μm. Click here for file

Additional file 3: Figure S2 Lack of focal CLN-5 immunostaining loss and perivascular cellularity in naïve spinal microvessels ***.*** Isosurface-rendered images generated from confocal z-stacks of 60 -μm thick cryosections from naïve mice showing continuity of CLN-5 staining (**green**) in naïve spinal microvessels. The lack of perivascular cellularity associated with typical inflamed microvessels is further highlighted with DRAQ5 staining for nuclei (**blue**). Scale = 20 μm. Click here for file
