# Supplementary figures and images for: Cell-selective knockout and 3D confocal image analysis reveals separate roles for astrocyte-and endothelial-derived CCL2 in neuroinflammation
Source: J Neuroinflammation. 2014 Jan 21;11:10. doi: 10.1186/1742-2094-11-10 (PMC3906899; doi:10.1186/1742-2094-11-10)

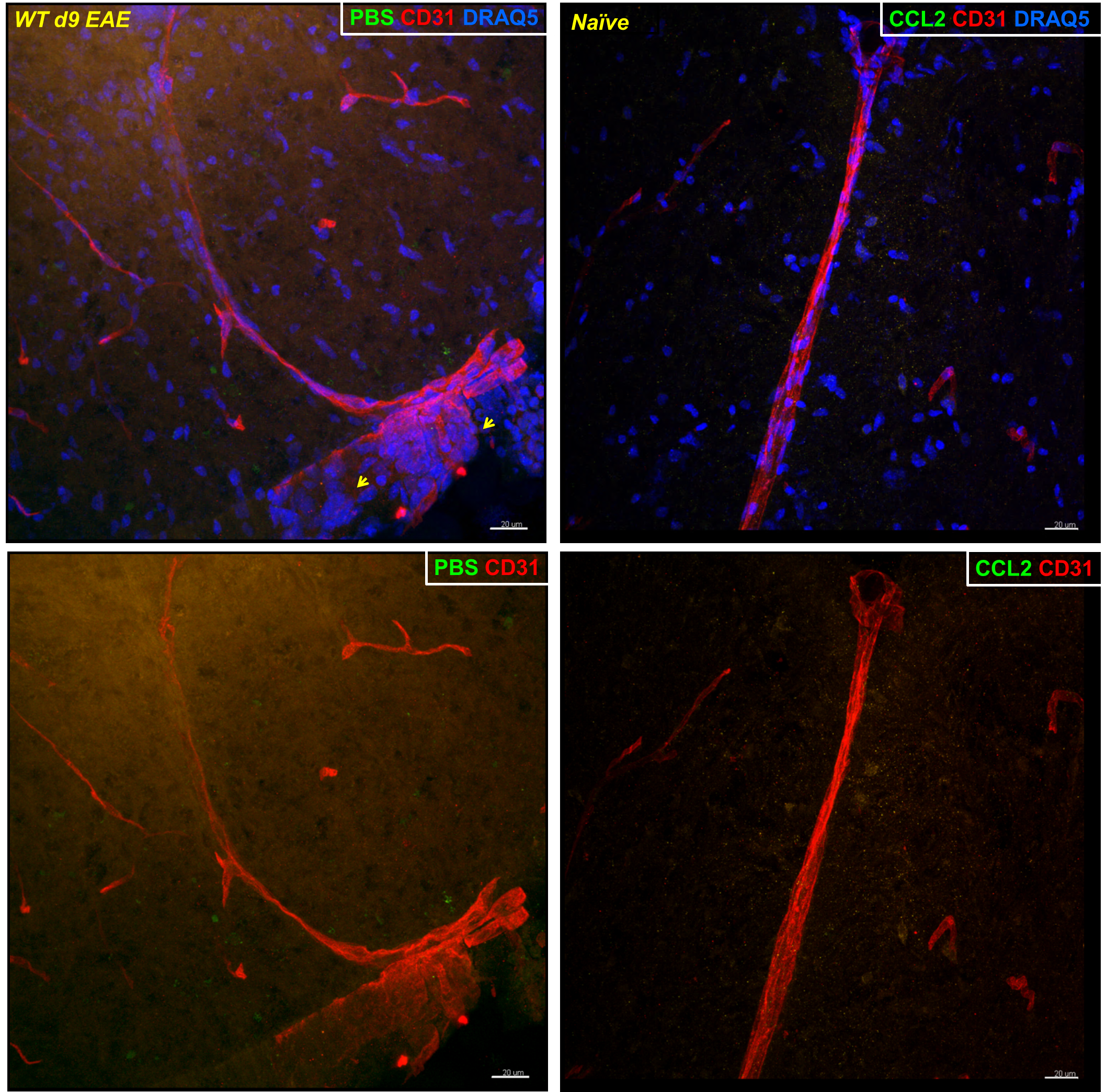

Supplement: Additional file 2: Figure S1 — Specificity of CCL2 immunostaining. Volume rendered images of z-stacks obtained from serialsections of d9 EAE spinal cords used in Figure 6 (left) and naïve (right) mice demonstrating specific immunoreactivity of the CCL2 antibody. No detectable CCL2 staining (green) was observed in naïve mice upon incubation with CCL2 antibody or in EAE mice in the absence of primary antibody. The endothelium is highlighted with CD31 (red), while DRAQ5 staining reveals the nuclei (blue). Scale = 20 μm. [file 1742-2094-11-10-S2.tiff]

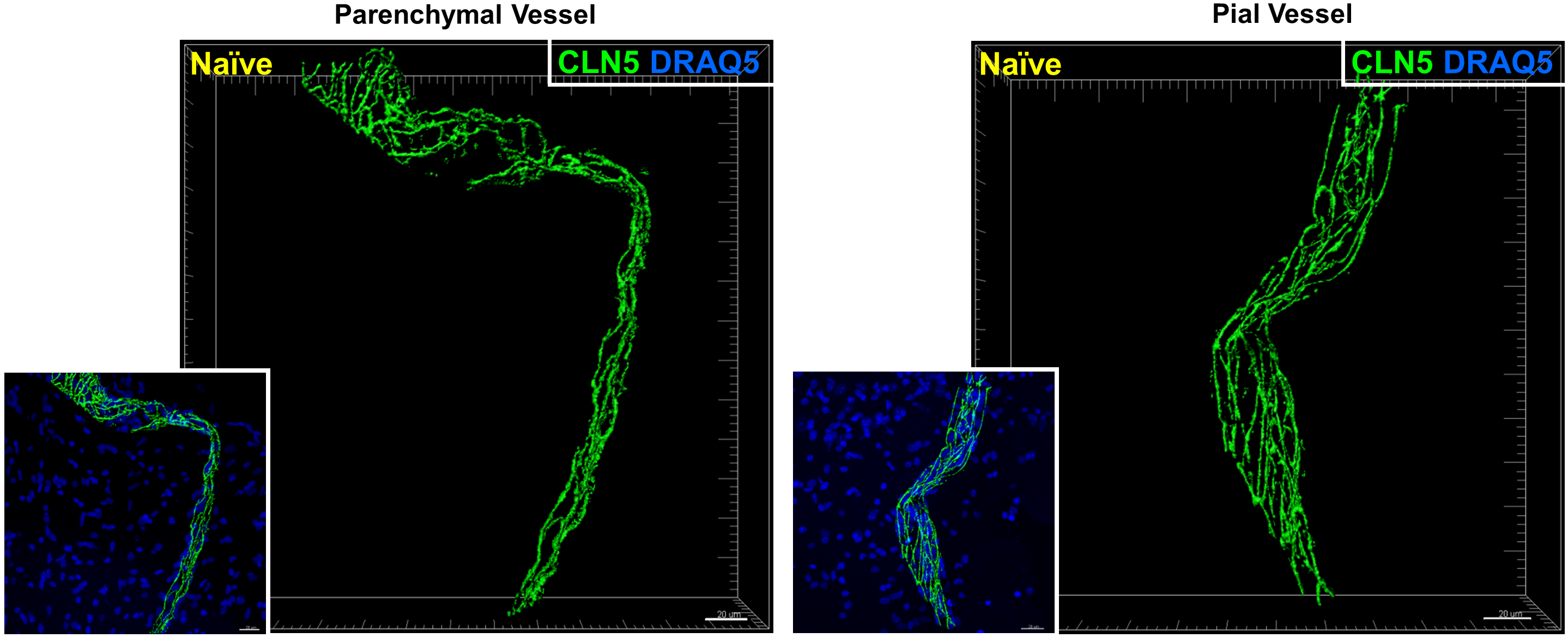

Supplement: Additional file 3: Figure S2 — Lack of focal CLN-5 immunostaining loss and perivascular cellularity in naïve spinal microvessels . Isosurface-rendered images generated from confocal z-stacks of 60 -μm thick cryosections from naïve mice showing continuity of CLN-5 staining (green) in naïve spinal microvessels. The lack of perivascular cellularity associated with typical inflamed microvessels is further highlighted with DRAQ5 staining for nuclei (blue). Scale = 20 μm. [file 1742-2094-11-10-S3.tiff]
